# Analysis of functional surfaces on the actin nucleation promoting factor Dip1 required for Arp2/3 complex activation and endocytic actin network assembly

**DOI:** 10.1016/j.jbc.2022.102019

**Published:** 2022-05-06

**Authors:** Su-Ling Liu, Heidy Y. Narvaez-Ortiz, Matt Miner, Jack Kiemel, Nicholas Oberhelman, April Watt, Andrew R. Wagner, Qing Luan, Luke A. Helgeson, Brad J. Nolen

**Affiliations:** Department of Chemistry and Biochemistry, Institute of Molecular Biology, University of Oregon, Eugene, Oregon, USA

**Keywords:** actin, arp2/3, endocytosis, WASP, Wsp1, nucleation, branching, ARM, armadillo repeat motif, EMM, Edinburgh minimal media, mGFP, monomeric GFP, NPF, nucleation promoting factor, TEV, tobacco etch virus, WDS, WISH/DIP/SPIN90

## Abstract

Arp2/3 complex nucleates branched actin filaments that drive processes like endocytosis and lamellipodial protrusion. WISH/DIP/SPIN90 (WDS) proteins form a class of Arp2/3 complex activators or nucleation promoting factors (NPFs) that, unlike WASP family NPFs, activate Arp2/3 complex without requiring preformed actin filaments. Therefore, activation of Arp2/3 complex by WDS proteins is thought to produce the initial actin filaments that seed branching nucleation by WASP-bound Arp2/3 complexes. However, whether activation of Arp2/3 complex by WDS proteins is important for the initiation of branched actin assembly in cells has not been directly tested. Here, we used structure-based point mutations of the *Schizosaccharomyces pombe* WDS protein Dip1 to test the importance of its Arp2/3-activating activity in cells. Six of thirteen Dip1 mutants caused severe defects in Arp2/3 complex activation *in vitro*, and we found a strong correlation between the ability of mutants to activate Arp2/3 complex and to rescue endocytic actin assembly defects caused by deleting Dip1. These data support a model in which Dip1 activates Arp2/3 complex to produce actin filaments that initiate branched actin assembly at endocytic sites. Dip1 mutants that synergized with WASP in activating Arp2/3 complex *in vitro* showed milder defects in cells compared to those that did not, suggesting that in cells the two NPFs may coactivate Arp2/3 complex to initiate actin assembly. Finally, the mutational data reveal important complementary electrostatic contacts at the Dip1–Arp2/3 complex interface and corroborate the previously proposed wedge model, which describes how Dip1 binding triggers structural changes that activate Arp2/3 complex.

Arp2/3 complex is a seven subunit protein assembly that nucleates actin filaments in response to cellular signals (1, 2). The complex has little or no activity on its own, and its nucleation activity must be stimulated by regulatory proteins called nucleation promoting factors (NPFs) (3). During activation by WASP proteins, the largest family of NPFs, Arp2/3 complex must bind WASP, a WASP-recruited actin monomer, and the side of a pre-existing actin filament before nucleation is triggered (4, 5, 6). The requirement for actin filaments ensures that Arp2/3 complex nucleates a branched filament. Consequently, WASP-mediated activation allows Arp2/3 complex to create an assembly of highly branched (dendritic) actin networks that are optimal for pushing against broad flat membranes in processes like cellular motility and endocytosis (7). However, the requirement for preformed filaments in WASP-mediated activation means that branching nucleation must be preceded by a seeding step, in which an initial actin filament is created to kick-start the assembly process.

WISH/DIP/SPIN90 (WDS) family proteins, a class of NPFs with different biochemical properties than WASP proteins, are thought to have unique roles in cellular actin assembly compared to WASP (8, 9, 10). Unlike WASP, WDS proteins trigger actin filament nucleation by Arp2/3 complex without a pre-existing actin filament (8). Therefore, activation of Arp2/3 complex by WDS proteins creates a linear actin filament rather than a branch (8, 9). Importantly, linear filaments created by WDS protein–activated Arp2/3 complex stimulate branching nucleation by WASP-bound Arp2/3 complex *in vitro* (11). Therefore, WDS proteins are thought to provide the initial (seed) filaments to stimulate WASP-mediated activation of Arp2/3 complex during branched actin assembly in cells. While early models postulated a clear demarcation of Dip1 as the initiator of branched networks and WASP as the propagator, recent data show that the two NPFs potently synergize to coactivate Arp2/3 complex without a pre-existing actin filament, suggesting that WASP could play a role in both propagation and initiation of branched actin networks (12).

How NPFs activate Arp2/3 complex for either initiation or propagation of branched actin networks has been an important question, and while many studies have focused on activation by WASP, recent structures of Arp2/3 complex bound to WDS proteins have revealed fundamental insights into the activation mechanism. For instance, a recent cryo-EM structure reveals a snapshot of Arp2/3 complex in a fully active state, in which it is bound to the *Schizosaccharomyces pombe* WDS protein Dip1 and anchored to the end of the linear filament that it nucleated (13). In addition, an X-ray crystal structure of the human WDS protein SPIN90 bound to Arp2/3 complex revealed an intermediate state along the activation pathway in which the NPF is bound but the complex still adopts an inactive conformation (14). Comparison of these structures revealed that two major conformational rearrangements occur upon activation. First, the two clamp subunits (ARPC2 and ARPC4) twist relative to each other, moving Arp2 and Arp3 into a position in which they mimic the short pitch helical arrangement of actin subunits within a filament (13). Second, Arp2 and Arp3 undergo subunit flattening, which switches them from a conformation similar to monomeric actin to one that mimics filamentous actin subunits (13, 15, 16). These conformational changes allow Arp2/3 complex to template the growth of the new filament. Biochemical data have demonstrated that both SPIN90 and Dip1 can stimulate at least one of these two structural changes: adoption of the short pitch conformation (8, 14). The structures revealed how WDS proteins could trigger this conformational switch; by binding to and bending a long alpha helix in the ARPC4 subunit (αD) and simultaneously wedging against its globular domain, Dip1 appears to trigger twisting of the entire clamp (Fig. 1*A* and Video S1), thus moving Arp2 into the short pitch arrangement next to Arp3. While the structures revealed several complementary interactions between ARPC4 and two key helices in Dip1 (H5-2 and H6-2), the importance of these interactions is uncertain and the so called wedge model for Dip1-mediated activation has not been tested.

**Figure 1:**
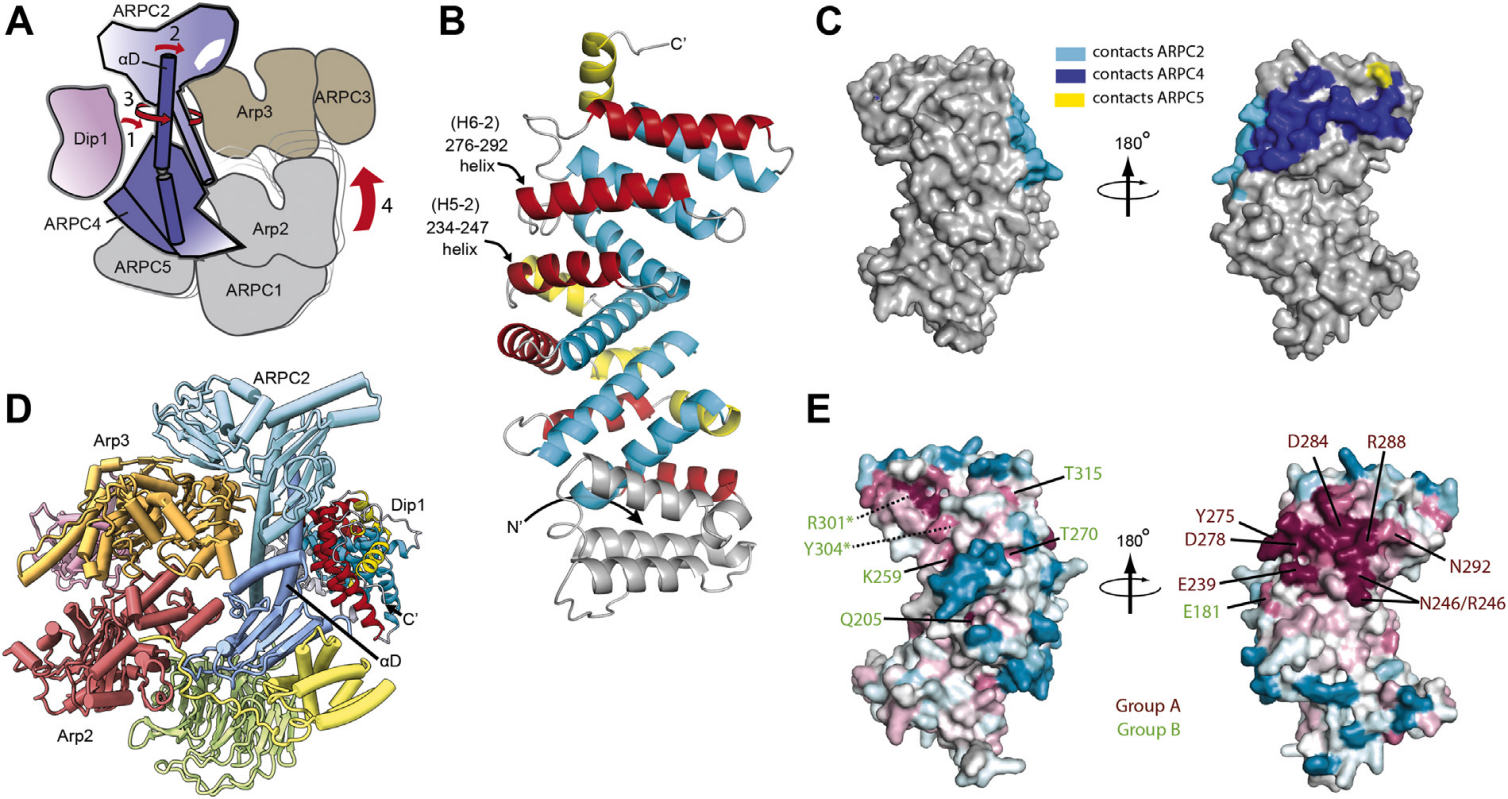
**Identification of conserved residues in Dip1.***A*, cartoon depiction of a model for Dip1-mediated activation of Arp2/3 complex. In this model, Dip1 binds to the long αD helix in ARPC4 and wedges against the globular portion of ARPC4 (step 1). This bends the αD helix (step 2), which then causes twisting of the clamp (step 3) and subsequent rotation of a rigid body of subunits that moves Arp2 into the short pitch arrangement next to Arp3 (step 4). *B*, ribbon diagram of AlphaFold model of full-length Dip1. Armadillo repeat motifs (ARM) are colored *yellow*, *red*, and *cyan* for helices 1, 2, and 3 within the repeat, respectively. The two alpha helices (H5-2 and H6-2) that contact Arp2/3 complex in high resolution structures are labeled. The nomenclature for the interacting alpha helices (H5-2 and H6-2) is based off of the repeat number and the helix number within the human WDS protein, SPIN90. Based on the AlphaFold model, Dip1 has one fewer ARM domain than SPIN90. *C*, surface representation of Dip1 showing atoms that are within 5 Å of any atom in ARPC2 (*blue*), ARPC4 (*cyan*), or ARPC5 (*yellow*) when bound to Arp2/3 complex. *D*, AlphaFold Dip1 model docked onto the portion of Dip1 visible in the cryo-EM structure (235–366) (13). *E*, surface representation of Dip1 with residues colored according to conservation (from most to least conserved, the colors are: *magenta*, *pink*, *gray*, and *cyan*). A homology model of Dip1(46–358) was used for this conservation analysis. WDS, WISH/DIP/SPIN90.

Because they can seed branching nucleation by WASP-bound Arp2/3 complex *in vitro*, WDS proteins were hypothesized to play a role in providing the first actin filaments to initiate the assembly of branched actin networks in cells (11). However, few experiments have addressed possible roles for WDS proteins in regulating Arp2/3 complex *in vivo.* In metazoans, Arp2/3 complex plays a role in the assembly of lamellipodial protrusions, where it nucleates branched actin filaments that push the membrane outward (17, 18, 19, 20). One study showed that knockdown of the mammalian WDS protein SPIN90 decreased PDGF-induced actin assembly in lamellipodial protrusions in Cos7 cells. Another study showed that embryonic fibroblasts derived from SPIN90 KO cells had defective motility and altered cell shape (10, 21). Both of these observations suggest SPIN90 may play a role in seeding branched actin filament assembly. However, SPIN90 (also known as WISH and DIP) has been implicated in many other cellular functions, including regulation of the activity of the formin class of actin filament nucleation and elongation factors (9, 22), modulating the interactions of dynamin (23), regulation of the actin disassembly factor cofilin (24), regulation of small GTPases (25, 26), and activation of N-WASP (27). Therefore, the relative importance of activation of Arp2/3 complex and actin filament network seeding by SPIN90 in cells is unknown.

In yeast, Arp2/3 complex is required for the assembly of actin networks that drive invagination of the plasma membrane during endocytosis (28). The fission yeast WDS protein, Dip1, localizes to endocytic sites—along with Wsp1, the WASP family NPF—before endocytic actin assembly begins (29). Approximately 2 to 4 s after Dip1 and Wsp1 arrive, dynamic patches of actin filaments assemble that drive membrane invagination (29, 30). Dip1 and Wsp1 begin to dissociate from the patches ∼2 to 4 s before actin filaments reach their peak concentration, with each NPF having a total lifetime of about 12 to 14 s (29, 30). Deletion of Dip1 causes a significant defect in actin assembly at endocytic sites; while Wsp1 still localizes to cortical puncta, it remains stalled at the cortex—in some cases, for hundreds of seconds—before actin assembles (29). These observations point to a potential role for Dip1 in seeding branched actin assembly; if Dip1 is required to provide the first seed filaments to trigger Wsp1-mediated activation of the complex, deletion of Dip1 would be expected to cause Wsp1 to stall at the cortex. However, like SPIN90, it is possible that Dip1 has multiple cellular functions, and it is unclear whether its ability to directly activate Arp2/3 complex is what allows Dip1 to control the timing of endocytic actin assembly. Therefore, it is unknown whether actin filament nucleation by Dip1-activated Arp2/3 complex is important for initiating branched actin network assembly in cells.

Here, we use the recent structures of WDS proteins bound to Arp2/3 complex to design point mutations in Dip1 that allow us to (a) identify residues that are important to trigger activation and (b) test whether the ability to activate Arp2/3 complex is important for Dip1 to control the timing of endocytic actin assembly. Of a total of thirteen mutations in Dip1, six completely blocked activation of Arp2/3 complex by Dip1 under the conditions that we tested. All of these critical residues were in the H5-2 and H6-2 helices in Dip1, which pack against the long alpha helix (αD) in the Arp2/3 complex subunit ARPC4. Analysis of the structures revealed that these six residues make different sets of complementary electrostatic interactions with the ARPC4 subunit depending on whether clamp twisting has occurred in the complex. Their dual interaction modes allow Dip1 to shift its binding register along the ARPC4 αD helix, and this shift appears to be critical for Dip1 to bend αD, trigger clamp twisting, and move Arp2 into the short pitch conformation. By quantitative live cell imaging of fission yeast, we found that Arp2/3 complex activation potency was strongly correlated with the ability of mutants to rescue the *dip1Δ* phenotype, strongly supporting a model in which activation of Arp2/3 complex by Dip1 creates seed actin filaments that initiate endocytic actin assembly. While a subset of mutants that were highly defective *in vitro* showed mild *in vivo* defects, these mutants potently coactivated Arp2/3 complex with Wsp1, suggesting that coactivation by these two NPFs is important for controlling the timing of endocytic actin assembly in yeast.

## Results

### Identifying functionally important residues in Dip1

To better understand how Dip1 functions in cells and *in vitro*, we used structural data to identify its functionally important residues. The structure of Dip1 bound to activated Arp2/3 complex on the end of an actin filament was recently solved by cryo-EM (13) and serves as important starting point. However, the structure shows density for only the C-terminal region (residues 235–366) of Dip1, and there are currently no structures of full-length Dip1 (residues 1–374). Therefore, we first examined the structural model of full-length Dip1 generated by AlphaFold (31). The model folds into an extended armadillo repeat motif (ARM) domain with six ARM repeats and an N-terminal helical bundle cap (Fig. 1*B*). The three C-terminal ARMs adopt a structure nearly identical to the C-terminal end of Dip1 bound to activated Arp2/3 complex in the recent cryo-EM structure (Fig. S1). The predicted fold of full-length Dip1 is similar to the previously solved structure of the human WDS family protein, SPIN90 (RMSD = 2.9 Å for 234 superposed Cα atoms) (Fig. S1, (14)). When the full-length Dip1 AlphaFold model is docked onto the activated Arp2/3 complex structure, the C-terminal portion of Dip1 packs against the ARPC4 subunit, also making minor contacts to ARPC2 and ARPC5 (Fig. 1*C*). The docked model, along with the cryo-EM structure (13), reveal that most of the contacting residues are contributed by two helices on one face of the Dip1 ARM domain, H5-2 and H6-2, which pack against the long αD helix in ARPC4 to form a three helix bundle (Fig. 1, *B*–*D*). In the docked model, the N-terminal half of Dip1 protrudes from the side of the clamp subunits (ARPC2 and ARPC4) and projects into solvent away from the actin filament–binding face of the complex (Fig. 1*D* and Video S1). The lack of predicted contacts between the N-terminal half of Dip1 and Arp2/3 complex is consistent with our previous biochemical data showing that the N-terminal 160 residues are dispensable for activation of Arp2/3 complex in pyrene actin polymerization assays (8).

To identify conserved residues, we manually inspected a sequence alignment of diverse WDS family proteins and a surface conservation map generated using the Consurf server (https://consurf.tau.ac.il/) (Figs. 1*E* and S2) (32). While conserved residues were, as expected, clustered at the surface of helices H5-2 and H6-2 (Fig. 1*E*), we also identified several conserved residues away from the binding interface hotspot that may be important for Dip1 function (Figs. 1*E* and S2). We mutated a total of 13 conserved residues to investigate their roles in activating Arp2/3 complex and to better understand the importance of conserved contacts at the Arp2/3-Dip1 interface. Seven of the mutated residues were at the Dip1–Arp2/3 complex interface (group A mutations) and six were not (group B mutations) (Fig. 1*E*).

### Residues at the binding hotspot are critical for Dip1-mediated activation of Arp2/3 complex *in vitro*

**Figure 2:**
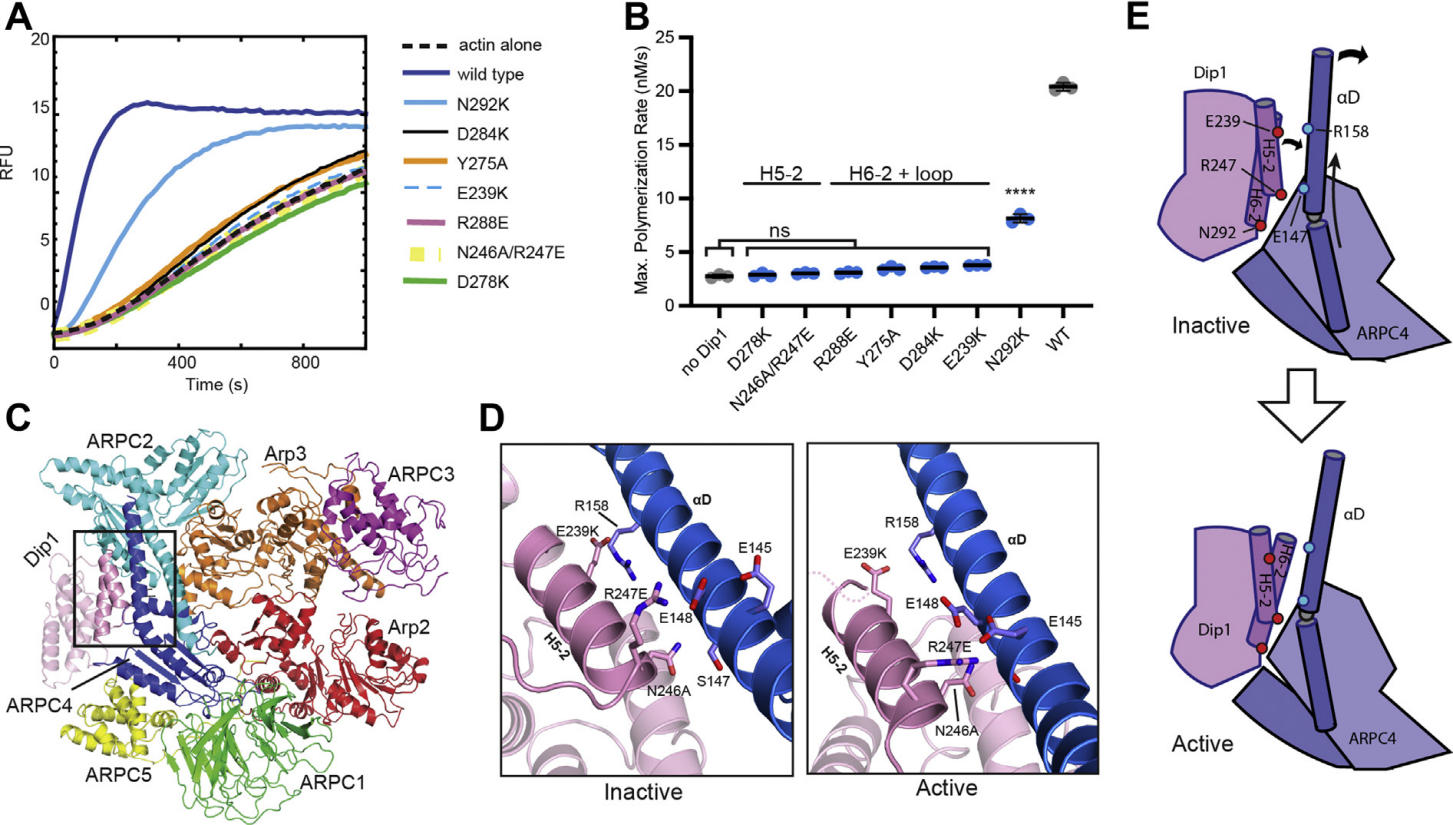
**Mutations at the Arp2/3 complex binding interface of Dip1 cause defects in Arp2/3 complex activation.***A*, polymerization time courses of 3 μM 15% pyrene-labeled actin and 50 nM SpArp2/3 complex with WT or mutant Dip1 proteins (5 μM). *B*, quantification of the maximum polymerization rate for reactions described in (*A*) run in triplicate. Error bars (when present) show SD. Absent error bars signify SD smaller than the data points. Statistical significance of difference between the mean rate for reactions with mutants *versus* reactions without Dip1 was assessed using a one-way ANOVA and *t* tests. n.s., non-significant; ∗∗∗∗*p* < 0.0001. *C*, cryo-EM model of Dip1 bound to Arp2/3 complex in the activated conformation (6W17). Box shows region highlighted in panel (*D*). *D*, close up of contacts between Dip1 helix H5-2 residues and Arp2/3 complex in the inactive (*left panel*) or active conformation (*right panel*). The *left panel* is hypothetical and shows the Dip1 AlphaFold model docked to a homology model of SpArp2/3 complex in the inactive conformation based on the X-ray crystal structure of SPIN90 bound to inactive *Bos taurus* Arp2/3 complex (6DEC). The image in the *right panel* was rendered from the cryo-EM model of Dip1 bound to activated Arp2/3 complex (6W17). *E*, depiction of the first two steps in the wedge model for Dip1-mediated activation of Arp2/3 complex. Dip1 binds the long helix in ARPC4 (αD), wedging against its globular portion, causing the helix to bend. The Dip1 and ARPC4 helices shift register as the ARPC4 helix bends, but most of the complementary electrostatic contacts are maintained (see Video S2).

As a first step in characterizing the Dip1 mutants, we expressed and purified them from *Escherichia coli* (Fig. S3) and tested their ability to activate *S. pombe* Arp2/3 complex in pyrene actin polymerization assays. We first tested mutants at the Arp2/3 complex–binding interface (on or near helix H5-2 and H6-2). Of the seven mutants in this group (group A), six had severe defects in their ability to activate Arp2/3 complex and at 5 μM did not show a significant increase in the maximum polymerization rate compared to reactions lacking Dip1 (Figs. 2, *A* and *B*, S4). One mutant, N292K, showed a moderate defect in its ability to activate Arp2/3 complex at 5 μM, decreasing the maximum polymerization rate to ∼40% of the rate observed with WT Dip1 (Figs. 2, *A* and *B*, S4).

To better understand how mutations at these positions influence activation, we examined their contacts with both the inactive (splayed) and active (short pitch) conformations of Arp2/3 complex, as differences in the inactive/active interfaces could explain how Dip1 binding shifts the complex toward the short pitch conformation (8). While the cryo-EM structure of Dip1-Arp2/3 complex on the filament end provides a snapshot of Dip1 bound to activated Arp2/3 complex, there are currently no structures of Dip1 bound to SpArp2/3 complex in the inactive conformation. We modeled this assembly by making a homology model of inactive *S. pombe* Arp2/3 complex using the inactive *Bos taurus* Arp2/3 complex as a template (4JD2). We then docked the AlphaFold model of full-length Dip1 onto the inactive SpArp2/3 complex using the SPIN90-bound BtArp2/3 complex (6DEC) as a guide (Fig. 2*D*, (14)).

Two of the six severely defective mutants (E239K and N246A/R247E) are on helix H5-2, which packs against the long αD helix in ARPC4. E239^Dip1^ is near the N terminus of helix H5-2 and makes an electrostatic interaction with R158 in ARPC4 in the inactive conformation of Arp2/3 complex but moves away from R158^ARPC4^ during activation (Fig. 2*D*). Residues N246A^Dip1^ and R247E^Dip1^ are at the C-terminal end of the H5-2 helix, where they interact with S147^ARPC4^ and E148^ARPC4^ in the αD helix, respectively, in the inactive state. Upon activation, N246^Dip1^ and R247^Dip1^ shift the register of their interaction with the αD helix, maintaining their interaction with S147^ARPC4^ and E148^ARPC4^ but making an additional interaction with Glu145^ARPC4^ further toward the N terminus of the αD helix. This register shift appears to be important in triggering bending of the αD helix, which is, in turn, linked to the twisting of the clamp (Fig. 2*E*). Charge reversal of E239^Dip1^ and R247^Dip1^ in these mutants likely disrupts these contacts, explaining the inactivity of these mutants.

**Figure 3:**
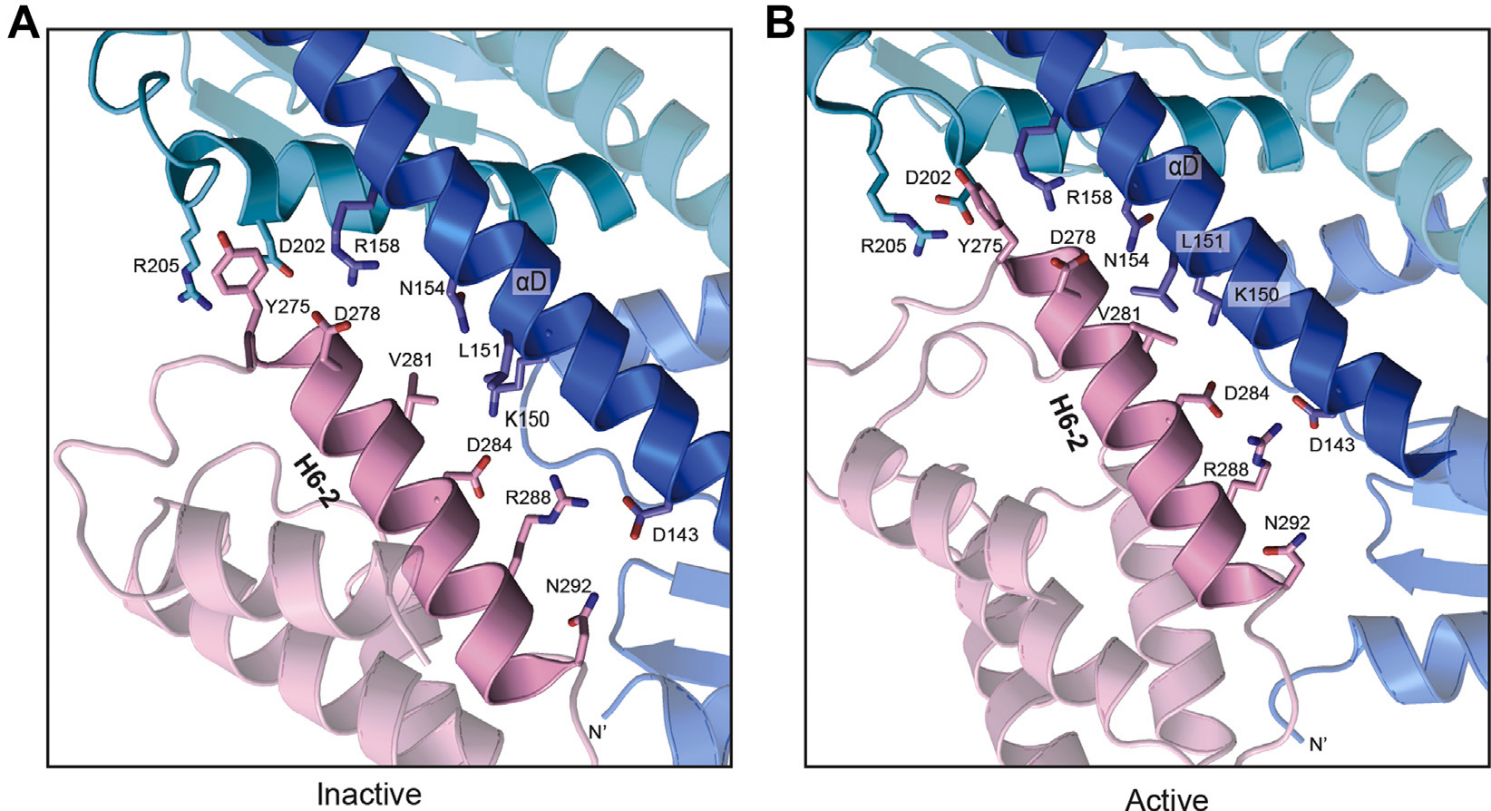
**Contacts between Dip1 helix H6-2 and Arp2/3 complex in the active and inactive states.***A*, close up of contacts between Dip1 helix H6-2 and Arp2/3 complex in the inactive state. This model was constructed by docking the AlphaFold model of Dip1 to SpArp2/3 complex as described in Figure 2*D*. *B*, structural model showing contacts between Dip1 helix H6-2 and Arp2/3 complex in the active state. The image was rendered from the cryo-EM model of Dip1 bound to active Arp2/3 complex (6W17).

Four of the six severely defective mutants in group A are either on helix H6-2 (D278K, D284K, and R288E) or in the loop between helix H6-1 and H6-2 (Y275A). Rotation and bending of the αD helix in ARPC4 closes a gap between the H6-2 and αD helices and changes the register of their interactions; so, these four residues show distinct interactions with inactive *versus* active Arp2/3 complex, though many of the complementary charged interactions are preserved in both states (Figs. 2*E* and 3). For instance, movement of H6-2 toward αD alters the geometry of the contacts but allows D284^Dip1^ and R288^Dip1^ to maintain complementary electrostatic interactions with ARPC4 αD helix residues K150^ARPC4^ and D143^ARPC4^, respectively. Y275^Dip1^ stacks against the aliphatic portion of R205^ARPC2^ in the inactive state, but upon activation, Y275^Dip1^ moves closer to ARPC4, making a cation–pi interaction with R158^ARPC4^. Loss of these interactions in the mutants explains their inactivity. Finally, while D278^Dip1^ interacts with the sidechain of R158^ARPC4^ in both the active and inactive states, its shifted register allows it to also contact N154^ARPC4^ in the active state. The D278K mutation would eliminate these interactions and likely causes electrostatic repulsion that reduces Dip1 binding and/or blocks bending of the ARPC4 helix.

The last mutation in category A (N292K) showed milder defects and at 5 μM increased the maximum polymerization rate to ∼8 nM/s under the conditions we tested, compared to ∼20 nM/s for 5 μM WT Dip1 and 2.8 nM/s for reactions without Dip1 (Fig. 2*B*). Residue N292K is at the C terminus of helix H6-2 (Fig. 3). This residue is either an N or D in most WDS protein sequences (Fig. S2). In both the inactive and active states, the C terminus of H6-2 packs against the N terminus of the ARPC4 subunit (Fig. 3). The interaction between Dip1 and the N terminus appears to be important in allowing Dip1 to serve as a wedge that bends ARPC4 αD and twists the clamp to move Arp2 into the short pitch conformation (Video S2). We speculate that mutation of this residue to a bulkier lysine residue disrupts this function, likely by mispositioning the N terminus of helix H6-2. We note that all of the mutations in group A are on the surface of Dip1; therefore, we do not expect that they influence the activity by causing misfolding of Dip1.

Together, our observations demonstrate that the interactions of conserved residues in helices H5-2 and H6-2 with the long αD helix in ARPC4 are critical for Dip1-mediated activation of Arp2/3 complex. Electrostatic complementarity is a key feature at the interface, and the ability to make interactions with multiple registers of the ARPC4 αD helix appears to be important to allow Dip1 to bend the αD helix and subsequently stimulate the major conformational change that moves Arp2 into the short pitch conformation (Fig. 2*E*).

### Conserved residues remote from the binding interface may allosterically influence Dip1–Arp2/3 interactions

We next tested the influence of conserved residues located away from the binding interface (group B). We reasoned that these residues may be conserved because they allosterically influence the interaction of Dip1 with Arp2/3 complex. Alternatively, they may be conserved for a function unrelated to Arp2/3 complex activation.

**Figure 4:**
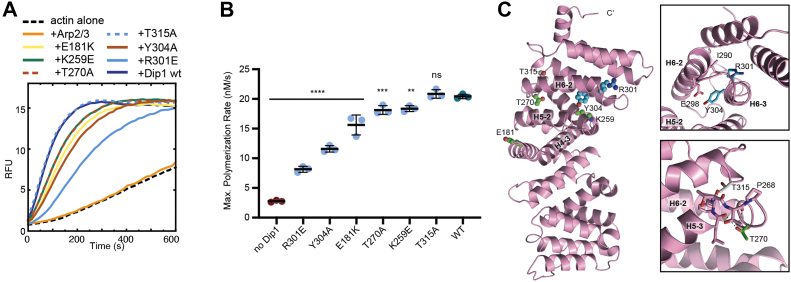
**Conserved residues in Dip1 may be allosterically connected to the Arp2/3 complex binding interface.***A*, polymerization time courses of 3 μM 15% pyrene-labeled actin and 50 nM SpArp2/3 complex with WT or mutant Dip1 proteins (5 μM). *B*, quantification of the maximum polymerization rate for reactions described in (*B*) run in triplicate. Error bars show SD. Statistical significance of difference between the mean rate for reactions with mutants *versus* reactions with WT Dip1 was assessed using a one-way ANOVA and *t* tests. n.s., non-significant; ∗∗*p*< 0.01; ∗∗∗*p*< 0.001; ∗∗∗∗*p* < 0.0001. *C*, cartoon diagram of Dip1 homology model (46–358) showing category B mutations colored by severity of defect in activating Arp2/3 complex; moderately defective, *cyan*; mild effect, *green*; no effect, *gray*. *Right panels* show close up of key interactions described in text.

The six mutants in group B are spread throughout the C-terminal half of Dip1. Five of the group B mutants (E181K, T270A, K259, R301E, and Y304A) moderately decreased activation of Arp2/3 complex by Dip1 (Fig. 4, *A* and *B*). Analysis of the structures revealed that four of these moderately inactivating mutations are linked to Arp2/3 complex interface through their contacts to Dip1 helices H5-2 and H6-2 (Fig. 4*C*). Specifically, R301^Dip1^ and Y304^Dip1^ are in helix H6-3 and pack against helix H6-2. We speculate that by making these contacts, R301^Dip1^ and Y304^Dip1^ influence the conformation of helix H6-2 and therefore its interactions with the long alpha helix in ARPC4. Similarly, E181^Dip1^ from helix H4-2 packs against the N terminus of helix H5-2 and may help position it to interact with ARPC4 (Fig. 4*C*). The T270A mutation lies in the H6-1 helix. T270^Dip1^ hydrogen bonds to the backbone of residues in the H5-3 helix and the loop preceding H6-1. Therefore, we speculate that mutation of this residue to alanine could allosterically influence the orientation of helix H6-2 by repositioning H6-1.

The fifth moderately defective mutant in group B, K259E, points toward the H4-3 helix on the side of Dip1 opposite from the Arp2/3-binding surface (Fig. 4*C*). We are currently unsure why this mutation (moderately) decreases Dip1-mediated Arp2/3 complex activation. Finally, one mutation in group B, T315A, had no influence on the activity of Dip1 in activating Arp2/3 complex. This residue is positioned in a loop between helix H6-3 and H7-1, pointed inward toward a set of hydrophobic residues (Fig. 4*C*). Analysis of the structure does not reveal a clear reason why this residue might be conserved. Together, our results on the group B Dip1 mutations reveal that most of the conserved residues away from the hotspot for Arp2/3 interactions likely influence the Dip1–Arp2/3 complex interface through allosteric interactions. We note that of the group B mutations, all but Y304A and R301E are on the surface. However, while Y304A and R301E are partly buried, CD spectra of these mutants were nearly identical to WT Dip1 (Fig. S5), indicating that defects caused by the Y304A and R301E mutations are not due to misfolding.

### Mutations at the Arp2/3 complex–binding interface cause defects in endocytic actin patches

To determine how conserved residues in Dip1 contribute to the assembly of actin filaments at endocytic sites, we created a subset of the group A and group B mutations in fission yeast DIP1. We deleted the entire DIP1 coding sequence with a URA cassette and integrated mutant *dip1* into the DIP1 locus, eliminating the URA cassette and leaving the mutant under control of its native promotor. To monitor the dynamics of endocytic actin patches in these strains, we tagged the actin binding and crosslinking protein Fim1 with mCherry and the *S. pombe* WASP family protein Wsp1 with monomeric GFP (mGFP) (30).

**Figure 5:**
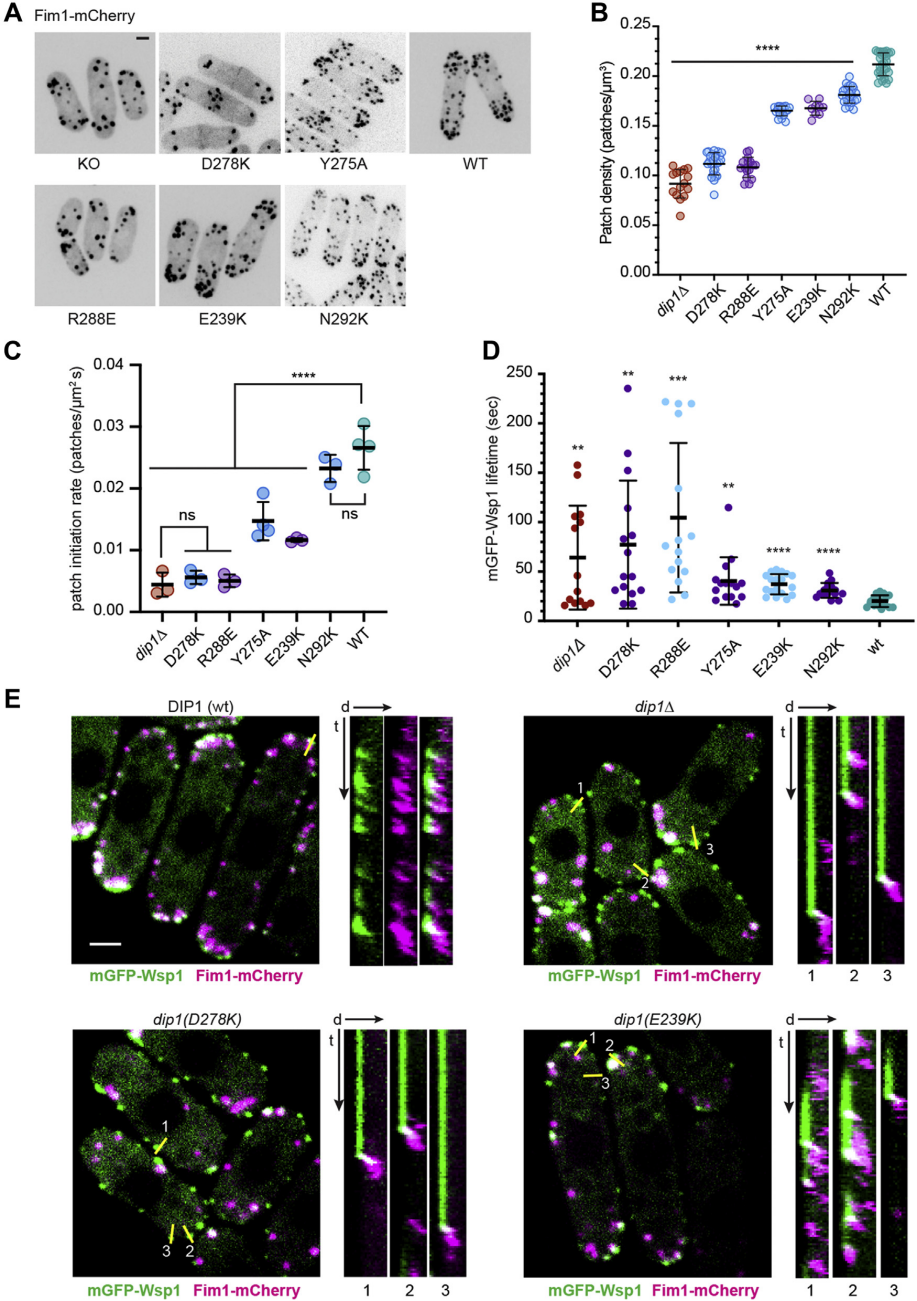
**Mutations at the Arp2/3 binding surface (group A) cause defects in endocytic actin patch assembly.***A*, spinning disk confocal images of WT and *dip1* mutant *S. pombe* cells expressing Fim1-mCherry. The images are maximum intensity projections from the full Z-stacks. The scale bar represents 2 μm. *B*, quantification of average endocytic actin patch density (expressed as number of patches per cell volume). Each average is calculated from at least nine independent images, with a single measurement corresponding to the average actin patch density in an image containing a cluster of 1 to 12 cells. Error bars: SD. An ANOVA test revealed the average densities were not equal. *Asterisks* indicate significance in one-tailed *t* test assuming unequal variances; ∗∗∗∗*p* ≤ 0.0001. *C*, rate of initiation of new endocytic actin patches for WT and *dip1* mutant *S. pombe*. Formation of new Fim1-mCherry-marked puncta was monitored over a 30 s interval. Each data point represents the initiation rate within one cell. An ANOVA test revealed the initiation rates were not equal. *Asterisks* indicate significance in one-tailed *t* test assuming unequal variances, with asterisks denoting *p* value range as described in panel (*B*). Error bars show SD. *D*, quantification of the lifetime of mGFP-Wsp1 cortical puncta lifetime plots for WT and *dip1* mutant cells. An ANOVA test revealed the average lifetimes were not equal. *Asterisks* indicate *p* values for a two-tailed *t* test assuming unequal variances, with asterisks denoting *p* value range as described in panel (*B*). *E*, spinning disk confocal microscopy images of WT and *dip1* mutant *S. pombe* expressing mGFP-Wsp1 and Fim1-mCherry. *Yellow lines* show single pixel line segments used to generate kymographs to the right of the main image. The scale bar represents 2 μm. Kymographs are oriented with distance (d) *arrow* pointing away from the cell edge and into the cytoplasm. The time arrow (*t*) is 60 s. mGFP, monomeric GFP.

We first examined the influence of five mutants in group A with significant defects in activating Arp2/3 complex *in vitro* (Y275A, R288E, E239K, N292K, and D278K) (Fig. 2). Previous data showed that *DIP1* deletion caused a reduction in the number of cortical puncta of Fim1, an actin filament–binding protein that marks the endocytic actin patches (29). Therefore, we compared the total number of Fim1 puncta in mutant cells to WT and KO cells. We expressed the patch count as a density (patches/μm^3^) to account for potential variability in patch number due to cell size differences. All five group A mutants we tested showed a reduced number of endocytic actin patches compared to the WT strain (Fig. 5, *A* and *B*). Mutants D278K and R288E showed the greatest reduction in actin patch density, with patch densities close to that of the *dip1Δ* strain. The Y275A, E239K, and N292K mutant strains showed intermediate patch densities with values closer to the WT strain than the *dip1Δ* strain. Collectively, these data show that all of the Dip1 mutants that showed a severe defect *in vitro* also exhibited at least a moderate defect in endocytic actin patch numbers in cells.

The reduced number of actin patches measured at a single time point in the mutant strains could be explained by a reduced rate of new actin patch initiation. It could also be caused by a reduced lifetime of endocytic actin patches once actin assembly begins. To distinguish between these possibilities, we directly measured the rate at which new patches were initiated in the mutant and WT strains. We found that four of the five group A mutants showed a reduced rate of actin patch initiation compared to the WT strain (Fig. 5*C*). Two of the category A mutants, D278K and R288E had patch initiation rates indistinguishable from the KO, while two (Y275A and E239K) had rates between the KO and the WT values. The *dip1(N292K)* strain had an average initiation rate indistinguishable from the WT.

In WT *S. pombe*, Wsp1 assembles into cortical puncta for a few seconds before activating Arp2/3 complex to begin assembling actin filaments that recruit Fim1 (30, 33). When actin filaments reach a peak accumulation, the actin patch moves into the cytoplasm and begins to disassemble (30, 33). The Wsp1 punctum moves inward slightly (∼200–300 nm) with the actin patch (33, 34), then disassembles (30). The total average lifetime of Wsp1 puncta in WT cells is ∼12 s (30). In *dip1* deletion cells, Wsp1 puncta remains at the cortex for much longer on average, presumably because actin assembly fails to initiate in the absence of a preformed actin filament (29). Therefore, we next asked whether the Dip1 mutations increased the lifetime of Wsp1 puncta. Of the five category A mutants tested in cells, all showed a statistically significant increase in the average lifetime of Wsp1 puncta compared to WT cells, consistent with a role for Dip1-mediated activation of Arp2/3 complex in preventing Wsp1 from stalling at the nascent endocytic sites (Fig. 5, *D* and *E*). While the E239K, N292K, and Y275A mutations caused only slightly longer Wsp1 puncta lifetimes than the WT, two mutants, D278K and R288E, showed dramatic increases in average Wsp1 puncta lifetimes. The *dip1(D278K)* and *dip1(R288E)* strains also showed a high variance in lifetimes, similar to the *dip1Δ* strain. Collectively, these data show that mutations of residues in Dip1 important for activation of Arp2/3 complex cause significant defects in endocytic actin patch assembly.

### Mutation of residues that caused moderate defects in Arp2/3 complex *in vitro* showed little or no defect *in vivo*

**Figure 6:**
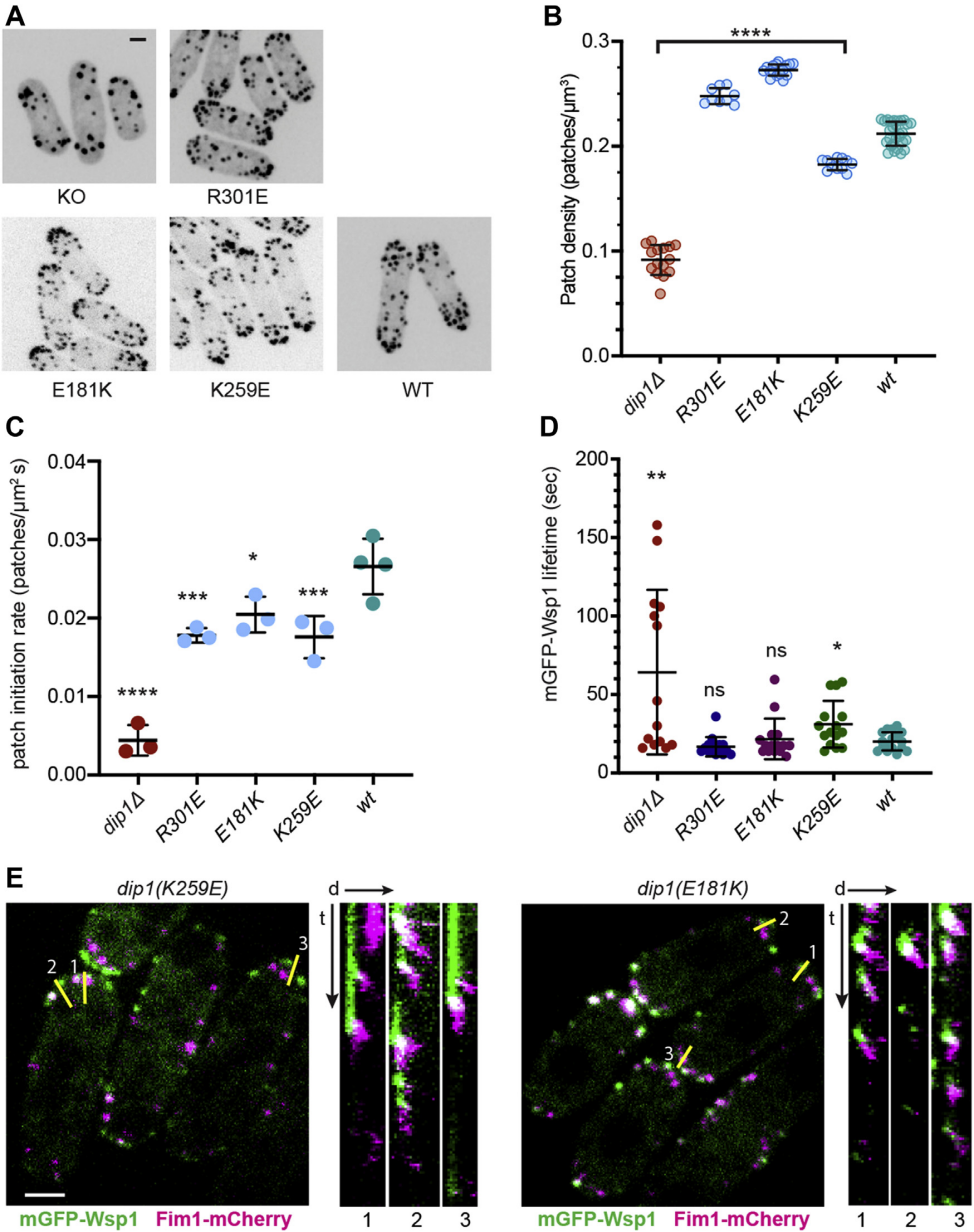
**Group B mutations have little or no influence on actin patch dynamics in cells.***A*, spinning disk confocal microscopy images of WT and *dip1* mutant *S. pombe* cells expressing Fim1-mCherry. The images are maximum intensity projections from the full Z-stacks. The scale bar represents 2 μm. Images of WT and *dip1Δ* (KO) control cells are taken from Figure 5*A*. We note that data from both group A and group B mutants were collected in the same imaging sessions as part of the same experiment. *B*, quantification of average endocytic actin patch density (expressed as number of patches per volume of cell) as described in Figure 5*B*. *C*, rate of initiation of new endocytic actin patches for WT and *dip1* mutant *S. pombe* as described in Figure 5*C*. *D*, quantification of the lifetime of mGFP-Wsp1 cortical puncta lifetime plots for WT and *dip1* mutant cells as described in Figure 5*D*. *E*, spinning disk confocal images of *dip1* mutant *S. pombe* expressing mGFP-Wsp1 and Fim1-mCherry. The scale bar represents 2 μm. *Yellow lines* show single pixel line segments used to generate kymographs to the right of the main image, as described in Figure 5*E*. mGFP, monomeric GFP.

We wondered whether mutation of the conserved residues away from the binding interface could also cause defects in actin assembly in cells, even though these mutants caused at most moderate *in vitro* defects. To test this, we integrated three group B mutations with moderate defects in activating Arp2/3 complex into yeast strains lacking WT *DIP1*, as described previously. We used all three metrics to determine if these mutations influenced seeding of endocytic actin patches: total patch number, patch lifetime, and mGFP-Wsp1 lifetime. In general, the influence of each of these mutations in cells was very mild. For instance, K259E showed only a small decrease in new patch initiation rate, a slight increase in average mGFP-Wsp1 lifetime, and a slight decrease in the number of endocytic actin patches compared to the WT strain (Fig. 6). The defects caused by the other two mutations we tested in this category (R301E and E181K) were also mild; while they each showed small decreases in the new patch initiation rate, they showed normal mGFP-Wsp1 puncta lifetimes and a slight increase in the number of endocytic actin patches compared to the WT strain (Fig. 6). The mild defects of the R301E mutation are especially surprising, given that this mutant showed over a ∼60% reduction in activity *in vitro* compared to WT Dip1 (Fig. 4). Overall, the mild defects of the group B mutants suggest that cells can tolerate all but the most significant reductions in Dip1 activity without incurring major defects in endocytic actin assembly.

### The *in vivo* activity of Dip1 mutants is correlated with their ability to activate Arp2/3 complex *in vitro*

**Figure 7:**
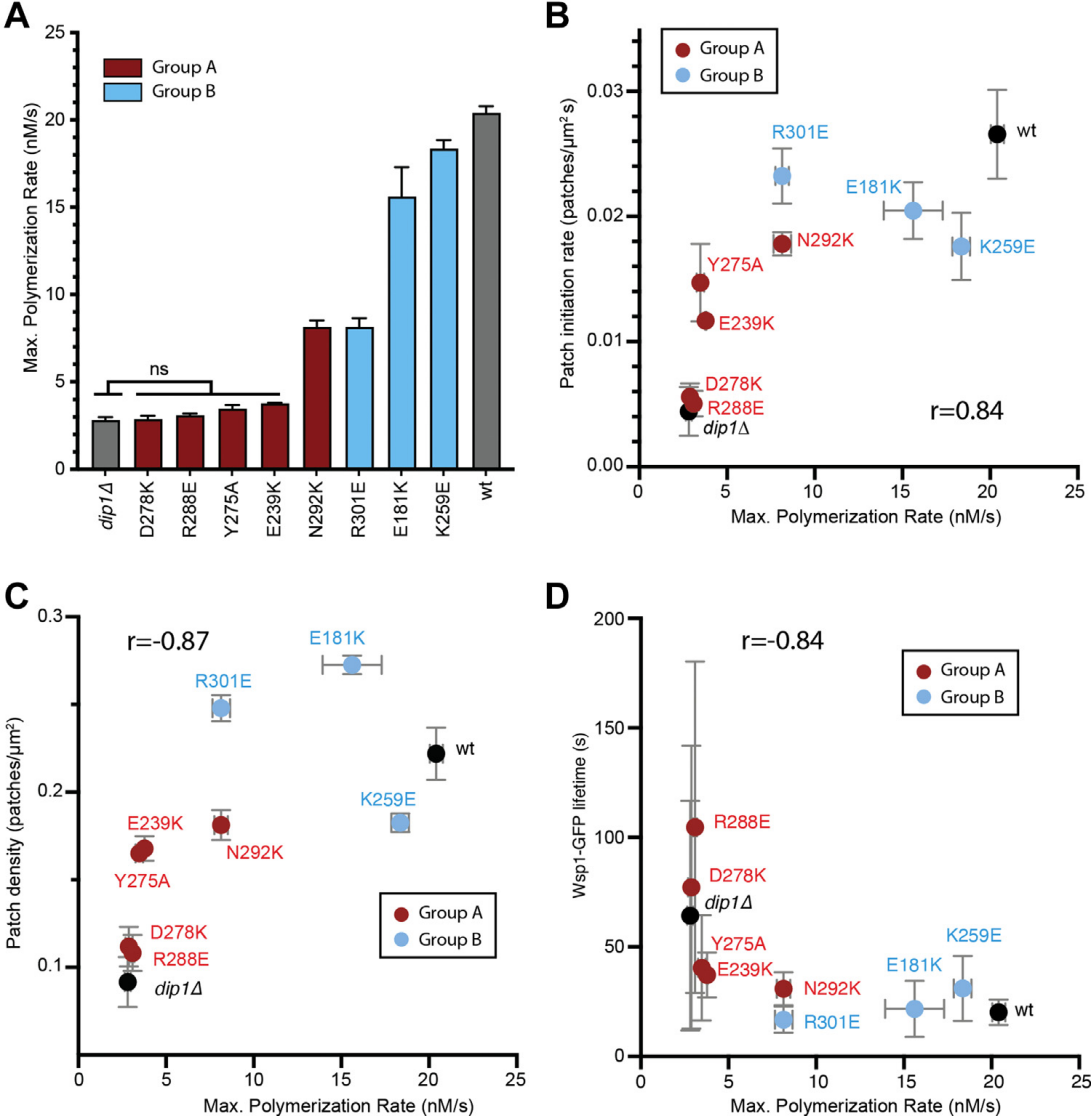
**The *in vivo* activity of Dip1 mutants correlates with their *in vitro* actin polymerization activity.***A*, average maximum polymerization rate for reactions containing each of the eight Dip1 mutants investigated *in vivo* taken from data described in Figures 2*B* and 4*B*. *B*, plot of average patch initiation rate *versus in vitro* maximum polymerization rate for WT and Dip1 mutants. The maximum polymerization rate of actin alone was plotted against the patch initiation rate for *dip1Δ*. *C*, plot of average actin patch density *versus in vitro* maximum polymerization rate for WT and *dip1* mutants. *D*, plot of average mGFP-Wsp1 lifetime *versus* maximum polymerization rate for WT and *dip1* mutants. The Spearman correlation coefficient is shown for panels (*B*–*D*). Error bars are as described for corresponding data in Figures 2*B*, 4*B*, 5, and 6. mGFP, monomeric GFP.

Based on the biochemical activities of Dip1 and the phenotype of *dip1Δ* cells (8, 11, 29), we hypothesized that the role of Dip1 is to activate Arp2/3 complex to create the first filaments that initiate endocytic actin patch assembly. However, it is possible that another function of Dip1 (other than activating Arp2/3 complex) is responsible for controlling the timing of actin assembly at endocytic sites. Our panel of mutants allowed us to address this question. Specifically, we reasoned that if the ability of Dip1 to activate Arp2/3 complex controls the timing of actin patch assembly in cells, that there should be a correlation between the severity of mutations on Arp2/3 complex activation and *in vivo* actin patch assembly defects. To assess this, we plotted the influence of each mutant on actin patch formation *versus* its influence on Arp2/3 complex activation *in vitro* (Fig. 7). These analyses show that mutations in group A are more defective than those in group B and reveal a nonlinear correlation between *in vitro* and *in vivo* activity. The correlation between the *in vitro* and *in vivo* activity strongly argues that Dip1 controls the timing of actin patch assembly through activation of Arp2/3 complex. While the plots in Figure 7 suggest a hyperbolic relationship between Dip1 activity *in vitro* and *in vivo*, we cannot eliminate the alternate possibility that there may be a threshold of Dip1 activity required for normal actin assembly dynamics.

### Wsp1 rescues the ability of most but not all Dip1 mutants to activate Arp2/3 complex

**Figure 8:**
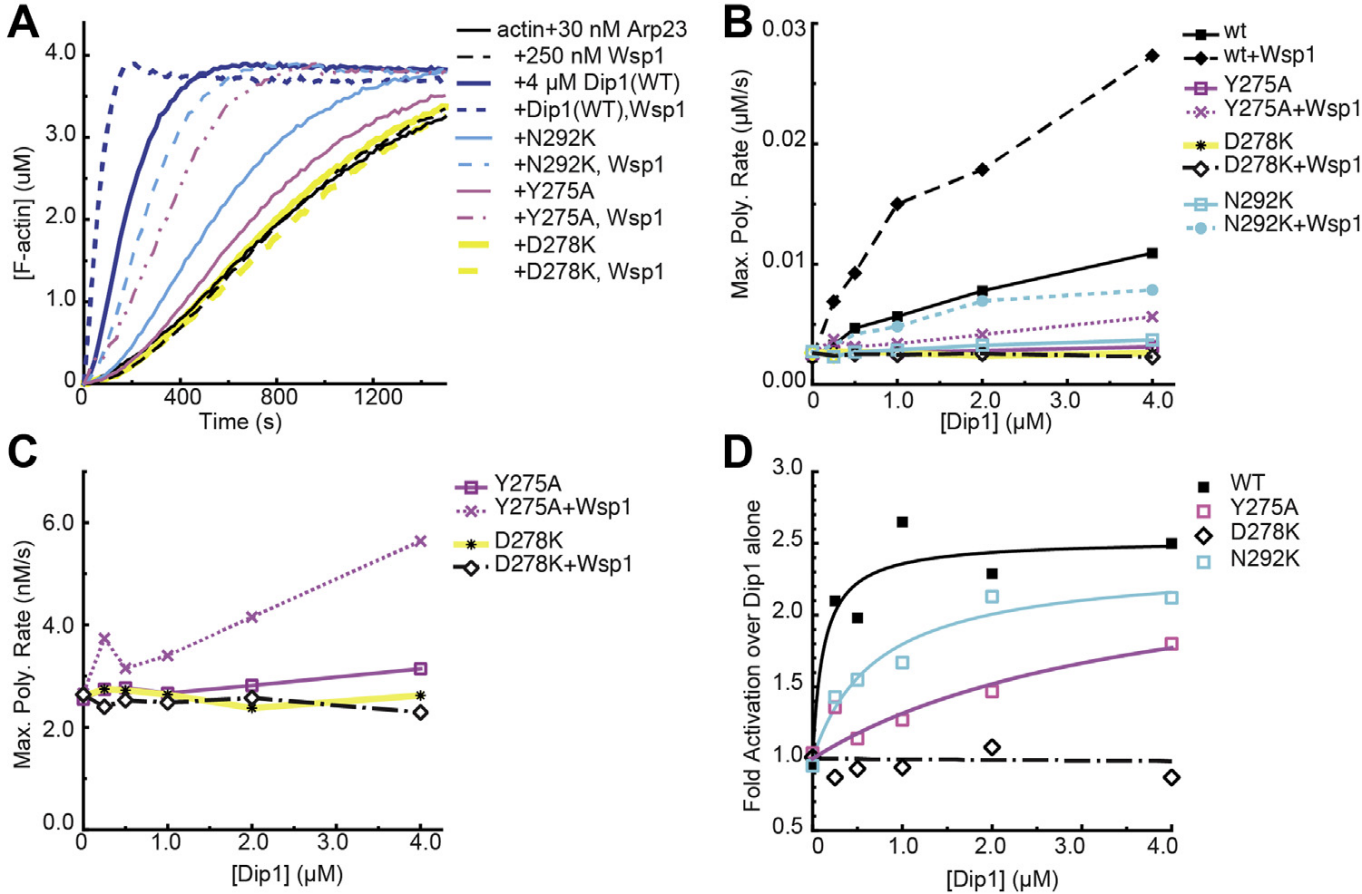
**Wsp1 rescues the ability of some Dip1 mutants to activate Arp2/3 complex.***A*, time courses showing polymerization of 3 μM 15% pyrene-labeled actin with WT or mutant Dip1 proteins (4 μM) and 30 nM SpArp2/3 complex with or without 250 nM Wsp1-VCA. At these concentrations, Wsp1-VCA alone shows negligible activation of Arp2/3 complex. *B*, maximum polymerization rate *versus* concentration of Dip1 for mutants shown in panel (*A*). Reactions contained 3 μM 15% pyrene-labeled actin, 30 nM SpArp2/3, 0 to 4 μM Dip1, and either 0 or 250 nM Wsp1-VCA. *C*, zoomed in view of plot in panel (*B*) showing the Y725A and D278K mutants with or without Wsp1-VCA. *D*, fold activation of Arp2/3 complex comparing reactions with both Dip1 and Wsp1-VCA to reactions with Dip1 but no Wsp1-VCA. Data were fit as described in the methods.

Despite the correlation between *in vitro* and *in vivo* activity, some mutants with very little or no activity *in vitro* showed greater *in vivo* activity than expected. For instance, the D298K and Y275A mutants both failed to activate Arp2/3 complex *in vitro*, but Y275A was less defective than D278K by all three measures of *in vivo* activity (Fig. 7). While there are multiple possible explanations for this behavior, one possibility is that cellular factors might rescue the activity of some Dip1 mutants so that their *in vivo* activity is closer to WT Dip1. Importantly, we recently showed that Dip1 activity is increased by Wsp1 *in vitro* and provided evidence that the two NPFs might synergize *in vivo* (12). Therefore, we asked if Wsp1 could rescue Arp2/3 complex activation by mutant Dip1 proteins. We found that low concentrations of Wsp1—which on their own were not enough to activate Arp2/3 complex—showed synergy with most but not all Dip1 mutants. For instance, addition of Wsp1 to the N292K Dip1 mutant increased the maximum polymerization rate approximately twofold over reactions without Wsp1, similar to the increase stimulated by adding Wsp1 to WT Dip1 (Fig. 8). The Y275A mutant, which shows barely detectable activity in the absence of Wsp1, also showed significant coactivation with Wsp1; addition of Wsp1 to 4.0 μM Dip1(Y275A) increased the maximum polymerization rate from 3.1 to 5.6 nM/s (Fig. 8). The partial rescue of Dip1 activity in the Y275A mutant by Wsp1 may explain why the Y275A mutant is not completely defective *in vitro*. In contrast, Wsp1 did not influence the activity of Dip1(D278K), consistent with our observation that the *dip1(D278K)* strain has a phenotype nearly identical to the Dip1 KO. Therefore, our data suggest that synergy between Wsp1 and Dip1 restores Arp2/3 activation by some Dip1 mutants to a level that supports normal actin assembly.

## Discussion

Here, we show that residues within a conserved surface patch on and around helices H5-2 and H6-2 on the *S. pombe* protein Dip1 are important for activation of Arp2/3 complex. Previously, two residues within this conserved patch (E239^Dip1^ and R288^Dip1^) were also shown to be important for SPIN90 (the human WDS protein) to activate Arp2/3 complex. Together, these observations suggest that activation of Arp2/3 complex is a conserved function of WDS proteins, which are present in broad range of organisms, from yeasts to metazoans*.* A key future direction will be to determine the roles of WDS-mediated activation of Arp2/3 in controlling actin network dynamics in different cellular contexts within higher eukaryotes. For instance, it will be important to understand whether activation of Arp2/3 complex is required for the reported roles of the human WDS protein SPIN90 in endocytosis and lamellipodial protrusion (10, 35). The mutations we describe here will be important tools in addressing these questions.

Our mutational data reveal that residues in helix H5-2 and H6-2 in Dip1 with electrostatic complementary to residues in ARPC4 αD are critical for activation of Arp2/3 complex. These data support the importance of the complementarity of the helices in the three helix bundle at the interface of Dip1 and Arp2/3 complex and are consistent with the wedge model of activation, in which helices from Dip1 slide against the ARPC4 αD helix to bend it, in turn causing ARPC4 and ARPC2 (the clamp subunits) to twist relative to one another to move Arp2 into the short pitch conformation (Figs. 1*A* and 2*E*, Video S2) (13). An important open question is how the flattened conformations of Arp2 and Arp3 are stimulated during WDS protein–mediated activation of Arp2/3 complex. Our current speculation is that stimulation of the short pitch conformation and flattening are weakly coupled conformational changes (36), so by triggering clamp twisting through the wedge mechanism, WDS proteins may stimulate both major activating conformational changes. However, additional work is required to address this question.

That many of the interactions made by the conserved charged residues at the Dip1–Arp2/3 complex interface appear to be maintained in both the inactive and active conformations of Arp2/3 complex was somewhat unexpected because of dramatic difference in the affinity of Dip1 to inactive *versus* active conformations of Arp2/3 complex. Specifically, while Dip1 binds relatively weakly to inactive Arp2/3 complex (8), lifetime analysis from single molecule total internal reflection fluorescence data suggests that it binds much more tightly to Arp2/3 complex on the end of the filament after it has triggered Arp2/3 complex to activate nucleation (37). The tighter binding to the activated complex ensures that unlike WASP proteins, which are released before nucleation (38), WDS proteins remain bound to Arp2/3 complex on the end of the nucleated filament and are thus consumed during the reaction (37). The single turnover nature of WDS proteins is thought to be important in limiting their activity within dendritic actin networks, since WDS-mediated activation of Arp2/3 complex creates linear rather than branched actin filaments (8, 37). We speculate that the increased Dip1 affinity for activated Arp2/3 complex is achieved by tighter packing of the H6-2 helix against ARPC4 αD in the activated state. This tighter packing generally improves the shape complementarity of the interaction surface, for example, by bringing hydrophobic resides into closer contact at the center of the three helix bundle and allowing Y275^Dip1^ to pack more tightly against ARPC4 (Fig. 3).

Here, we found that some residues in Dip1 that do not contribute to the Arp2/3 complex–binding site are nonetheless important for activation. In particular, we identified four conserved residues that appear to be important for activation of the complex because they help position the H5-2 and H6-2 helices, thereby allosterically influencing the Arp2/3 complex–binding site. This finding is important because it reveals a structural mechanism by which Dip1-mediated activation of Arp2/3 complex could be regulated; controlling the position of H5-2 and H6-2 could influence the interaction of Dip1 with the ARPC4 αD helix and consequently allow a regulator of Dip1 to tune its activity toward the complex. Some evidence already points to such a mechanism for SPIN90. Specifically, structural data show that the H5-2 and H6-2 helices are positioned differently comparing inactive to active N-terminal truncations of SPIN90 (14). However, these truncations contain different lengths of polypeptide N-terminal to the ARM domain (which contains the conserved patch of Arp2/3 complex–contacting residues), and the currently available structural and biochemical data do not reveal how regions outside the ARM domain might influence the conformation of H5-2 and H6-2. Furthermore, it is important to note that while we speculate that residues away from the Arp2/3 complex–binding surface can allosterically influence the positions of the two helices that contact Arp2/3 complex (H5-2 and H6-2), we cannot rule out other potential mechanisms for the effect of these residues.

The correlation between the ability of Dip1 mutants to activate Arp2/3 complex *in vitro* and their ability to rescue the defects in actin assembly in a *dip1Δ* background strongly argues that Dip1-mediated activation of Arp2/3 complex is important for controlling the timing of actin assembly at sites of endocytosis. Together with previous data showing the biochemical ability of Dip1 to seed branched actin filament nucleation (11), these observations support a model in which Dip1 provides the first seed filaments to initiate the assembly of endocytic actin networks. We note that Dip1 is not the sole source of seed filaments because in the *dip1Δ* strain initiation of new actin patches is slowed but not completely halted, indicating that a different seed-generation mechanism is also at play. Several other potential seeding mechanisms have been reported. For instance, in *S. pombe*, the actin filament–binding protein cofilin severs filaments in pre-existing endocytic actin patches, and the liberated fragments are thought to diffuse to new endocytic sites to seed branching nucleation (39). This severing diffusion mechanism cannot fully explain patch initiation events observed in the absence of Dip1, as it depends on other seeding mechanisms to generate actin patches that can be acted on by cofilin. Other potential seed generation mechanisms include nucleation by formin proteins (40), Arp2/3-independent nucleation activity of WASP family proteins (41), and priming by an Arp1/11-seeded mini filaments (42), though none of these mechanisms are known to function in fission yeast.

Our data showed that only the most defective Dip1 mutants *in vitro* showed substantial defects in endocytic actin assembly in cells. While it is possible that the activity of all but the most defective mutants are rescued in cells by synergizing with Wsp1, another possibility is that the initiation of new endocytic actin patches is only moderately sensitive to decreased actin filament seed generation by Dip1. Knowledge of the filament architecture at endocytic sites will likely be key to addressing how many seed filaments are required to generate functional endocytic actin networks. Previous electron microscopy images have shown that budding yeast endocytic sites are likely highly dendritic with few linear actin filaments (43), suggesting few linear seed filaments are required for initiation. In fission yeast, fluorescence-based measurements indicate that an average of ∼20 Dip1 molecules and ∼300 Arp2/3 complexes are present at peak concentrations (29, 30, 33). Given the single turnover nature of Dip1 (37), this suggests a maximum of 20 linear actin filaments, less than 10% of the total filaments generated assuming each Arp2/3 complex nucleates one filament. Some evidence suggests that branches also dominate at sites of endocytosis in mammalian cells. For example, platinum replica staining of endocytic sites in mouse B16F1 cells showed highly dendritic actin at endocytic sites (44). However, recent cryo-EM tomograms showed endocytic actin networks in human SK-MEL-2 cells consist of an approximately equal mix of linear and branched actin filaments (45). We anticipate that future studies will be aimed at determining not only the proportion of linear actin filaments at endocytic sites but whether linear actin filaments at these sites have a role beyond branched actin network initiation.

We showed here that a Dip1 mutant (D278K) that was severely defective activating Arp2/3 complex on its own or with WASP showed defects similar to the DIP1 KO in cells. In contrast, the Y275A mutant that showed little activity on its own but that synergized with WASP *in vitro* was far less defective *in vivo*. These observations support a previously proposed model in which Wsp1 and Dip1 synergize in cells to initiate the assembly of endocytic actin networks (12). Whether synergy between Wsp1 and Dip1 might be required for initiation of endocytic actin networks or if Dip1 can also initiate new actin patch assembly on its own is an important open question. A requirement for synergy with Wsp1 could be advantageous in that it would provide a simple signaling pathway to trigger Dip1 activity; factors that activate the NPF activity of Wsp1 would—through synergy—activate Dip1, without the requirement to activate a separate Dip1-specific activation pathway (12). In this way, branched actin network initiation and propagation would be coordinated through a single signaling pathway. However, our previous cell biological data show that deletion of the Wsp1 CA segment, which likely blocks Wsp1 from synergizing with Dip1 (12), decreases the rate of initiation of new actin patches, but not to the same extent as deletion of DIP1. Whether this means Dip1 has some ability to initiate new actin patches on its own or whether Dip1 synergizes with one of the other NPFs at endocytic sites (*e.g.*, MyoI, (46)) will be important to determine.

## Experimental procedures

### Cloning and protein purification

Dip1 mutants were constructed by amplifying pGV67-SpDip1, a plasmid for expression of full-length glutathione-*S*-transferase (GST)–tobacco etch virus (TEV)–Dip1 in *E. coli* (8), with nonoverlapping 5′-phosphorylated primers containing the mutation of interest and ligating and transforming the resulting PCR product. Protein expression was carried out by transforming the mutated Dip1 plasmids in BL21-CodonPlus(DE3)RIL *E. coli* and growing to an *A*_600_ of 0.6 to 0.7 in LB plus 100 μg/ml ampicillin and 35 μg/ml chloramphenicol at 37 °C before adding 0.4 mM IPTG and growing overnight at 22 °C for 12 to 14 h. Before harvesting, 2 mM EDTA and 0.5 mM PMSF were added to each culture. Cultures were centrifuged at 4000 rpm in a Fiberlite F8B rotor at 4 °C to pellet cells. Pelleted cells were resuspended in lysis buffer (20 mM Tris pH 8.0, 140 mM NaCl, 2 mM EDTA, 1 mM DTT, 0.5 mM PMSF, plus 2 protease inhibitor tablets (Roche)) and lysed by sonication before clarifying by centrifugation in a JA-20 rotor at 18,000 rpm for 30 min. The supernatant was loaded onto a glutathione sepharose column equilibrated in GST-binding buffer (20 mM Tris pH 8, 140 mM NaCl, 2 mM EDTA, and 1 mM DTT) and eluted with elution buffer (20 mM Tris pH 8.0, 140 mM NaCl, and 50 mM glutathione adjusted to pH 8.0). Peak fractions were pooled and TEV protease was added at a 25:1 ratio (by mass). After overnight dialysis against 20 mM Tris pH 8.0, 50 mM NaCl, and 1 mM DTT, the sample was loaded onto a 6 ml Resource Q column at pH 8.0 and eluted with a gradient of 50 mM to 500 mM NaCl. Peak fractions were concentrated in an Amicon-Ultra concentration device and loaded onto a Superdex 200 HiLoad 16/60 gel filtration column equilibrated in 20 mM Tris pH 8.0, 50 mM NaCl, and 1 mM DTT. Pure fractions were pooled and concentrated, and the final concentration was determined by measuring the absorbance at 280 nm (E_280_ = 36,330 M^-1^ cm^-1^ for all mutants and WT). Proteins were flash frozen in liquid nitrogen and stored at −80 °C. A subset of the Dip1 mutants were analyzed by CD spectroscopy to ensure mutations did not cause protein to unfold. Protein was dialyzed in 10 mM Na-phosphate pH 8.0 and 25 mM NaCl and diluted to 18 to 25 μM before taking spectra on a Jasco J-815 CD spectrometer.

*S. pombe* Arp2/3 complex was purified from the TP150 strain. Ten milliliters of turbid culture were transferred to 1 l of YE5S and grown at 30 °C overnight with shaking. In the morning, an additional 70 g of YE5S was added to each 1 l culture. At *A*_600_=6.0, 10 mM EDTA and 5 mM PMSF were added and cultures were centrifuged at 6500 rpm in a JLA10.5 rotor, washed with cold lysis buffer (20 mM Tris pH 8.0, 50 mM NaCl, 1 mM EDTA, and 1 mM DTT), and repelleted. All subsequent steps were carried out at 4 °C. Pellets were resuspended in lysis buffer (2 ml per gram of wet cell pellet) plus six protease inhibitor tablets (cOmplete Mini; Roche) per liter of lysis buffer. Cells were lysed by five passes through a Microfluidics Model M-110EH-30 Microfluidizer Processor at 23 kPSI. PMSF was added to 0.5 mM, and the lysate was clarified by centrifugation (JA-10 rotor at 9000 rpm for 25 min). The supernatant was then respun at 34,000 rpm for 75 min at 4 °C in a Fiberlite F37L rotor and filtered through cheesecloth. Ammonium sulfate was added under heavy stirring (0.243 g per ml of supernatant), and after 30 min, the solution was centrifuged in the Fiberlite F37L rotor at 34,000 rpm for 90 min. The pellet was resuspended in PKME (25 mM Pipes pH 7.0, 50 mM KCl, 3 mM MgCl_2_, 1 mM EGTA, 1 mM DTT, and 0.1 mM ATP) and dialyzed against PKME overnight before spinning at 34,000 rpm for 90 min in the F37L rotor. The supernatant was loaded on a column of GS4B beads precharged with GST-N-WASP-VCA and equilibrated in PKME (47). The column was washed with PKME +150 mM KCl until no protein was detected in the flow through by Bradford assay. Protein was eluted with PKME + 1 M NaCl into ∼2 ml fractions. Peak fractions containing Arp2/3 complex were pooled and dialyzed against 2 l of QA buffer (10 mM Pipes, 25 mM NaCl, 0.25 mM EGTA, 0.25 mM MgCl_2_, pH 6.8 w/KOH) in 50,000 molecular-weight cutoff dialysis tubing overnight. The complex was then loaded on a 1 ml MonoQ column and eluted with a linear gradient of QA buffer to 100% QB buffer (10 mM Pipes, 500 mM NaCl, 0.25 mM EGTA, 0.25 mM MgCl_2_, pH 6.8 w/KOH). Fractions containing Arp2/3 complex were pooled and dialyzed against 20 mM Tris pH 8.0, 50 mM NaCl, and 1 mM DTT overnight and then concentrated in Sartorius Vivaspin Turbo concentrator. The concentrated protein was loaded on a Superdex 200 size-exclusion column equilibrated in 20 mM Tris pH 8, 50 mM NaCl, and 1 mM DTT. Fractions with pure Arp2/3 complex were concentrated, and the final concentration was determined by measuring the absorbance at 290 nm (E_290_ = 139,030 M^-1^ cm^-1^) before flash freezing. Rabbit skeletal muscle actin was purified from muscle acetone powder (Pel-Freez 41995) and labeled with pyrene as previously described (12).

Wsp1-VCA was purified from bacteria as a GST-tagged fusion protein. The GST tag was removed with TEV protease. Briefly, BL21(DE3)-RIL cells were transformed with a pGV67-GST-Wsp1-VCA plasmid and grown in LB plus 100 μg/ml ampicillin and 35 μg/ml chloramphenicol at 37 °C to an *A*_600_ of 0.4 to 0.6 before inducing with 0.4 mM IPTG per liter of culture. After expressing protein at 22 °C for 12 to 14 h, EDTA and PMSF were added to 2 mM and 0.5 mM, respectively, before pelleting at 4000 rpm for 20 min at 4 °C in the Fiberlite F8B rotor. The pellet was resuspended in lysis buffer (20 mM Tris pH 8, 140 mM NaCl, 1 mM DTT, and 0.5 mM PMSF) plus two protease inhibitor tablets before sonication and clarification. The supernatant was loaded onto a GS4B column and washed before adding TEV protease and incubating with nutation at 4 °C overnight. The solution recovered from the column was diluted in 20 mM Tris pH 8.0, 2 mM DTT, and 2 mM EDTA to bring the NaCl concentration to 100 mM. The resulting solution was loaded onto a MonoQ 5/50 GL column and eluted with a gradient of NaCl (100–500 mM). Peak fractions containing Wsp1-VCA were concentrated and loaded onto a Superdex 75 size-exclusion column equilibrated in 20 mM Tris pH 8.0, 150 mM NaCl, and 1 mM DTT. Pure fractions were concentrated, and the final concentration was determined using an extinction coefficient of 5500 M^-1^ cm^-1^ before flash freezing in liquid nitrogen. The final purified product contains residues 497 to 574 of Wsp1 plus two amino acids (GS) appended to the N terminus left over from the TEV cut site.

### Pyrene actin polymerization assays

To measure time courses of actin polymerization, 2 μl of 5 mM MgCl_2_ and 20 mM EGTA was added to 20 μl of 15% pyrene-labeled actin monomers in G-buffer (2 mM Tris pH 8.0, 0.2 mM ATP, 0.5 mM DTT, 1 mM sodium azide, and 0.2 mM CaCl_2_) in a 96-well plate. Polymerization was initiated 1 to 2 min later by adding 78 μl of a solution containing other proteins, buffers, and salts to yield a reaction solution containing 10 mM imidazole pH 7.0, 50 mM KCl, 1 mM EGTA, 1 mM MgCl_2_, 0.2 mM ATP, and 1 mM DTT. Fluorescence measurements were made on a Tecan Safire2 plate reader at 15 s intervals using an excitation wavelength of 365 nm and an emission wavelength of 407 nm. The maximum rate of polymer formation was calculated by determining the maximum slope of each polymerization curve and converting relative fluorescence unit/second to nanomolar actin/second assuming the total amount of polymer at equilibrium is equal to the total concentration of actin minus 0.1 μM, the critical concentration.

Data in panel 8D were fit using the following equation:

$$Fold\;activation\left(FA\right)\;=\;FA_{min}+\left(FA_{max}-FA_{min}\right)\frac{\left[Dip1\right]}{\left[Dip1\right]+K_{1/2}}$$

### Construction of fission yeast strains

A URA4 DNA cassette that deleted the entire DIP1 coding region was amplified from KS-URA4 (48) using long primers complementary to the 5′ and 3′ UTR of DIP1 and transformed into VS1124A (a WT *S. pombe* stain in which Wsp1 is marked with an N-terminal mGFP and Fim1 is labeled with a C-terminal mCherry) using a lithium acetate transformation protocol to create the *dip1Δ* strain, SpBN229. To create Dip1 point mutations, DIP1 plus 200 nucleotides upstream and 200 nucleotides downstream were amplified from a *S. pombe* genomic DNA preparation and cloned into the pJK148 vector to produce vector Sp122. Point mutations of DIP1 were made using the same strategy as described for bacterial expression. Vectors with mutant *dip1* were then used as a template to amplify a knock-in cassette that replaces the URA cassette at the DIP1 locus in the QL002 strain. The knock-in cassettes were transformed into SpBN229 and selected by failure to grow on Edinburgh minimal media (EMM)–URA. Potential positive transformants were tested by amplifying the junctions and by sequencing the ORF to ensure the mutation was present. See Table S1 for a list of all strains used.

### Microscopy

*S. pombe* cultures were inoculated and grew in YE5S broth at 30 °C overnight with shaking to reach late log phase. The cultures were then diluted to *A*_600_ ≅ 0.05 with YE5S every 9 to 12 h and grown at 30 °C with shaking for 24 h. Cells were harvested at *A*_600_ ≅ 0.5, washed with EMM medium once, resuspended in EMM containing 10 mM propyl gallate, and then loaded on 25% gelatin/EMM gel pad for imaging. Gelatin pads were prepared by mixing 0.25 g of gelatin with 1 ml of EMM(5S) and melting in a heat block at 65 °C for 10 min. Gelatin mix was pipetted (100–200 μl) onto a glass slide and sealed by placing a second glass slide on top and fastening with binder clips. After cooling for 10 min, the slides were pried apart and 5 μl of cell suspension was added to the pad. A coverslip was sealed on top of the sample using a 1:1 mixture of petroleum jelly and lanolin.

Samples were imaged on a Nikon TE-2000 inverted microscope equipped with a Wallac Ultraview CSU-10 spinning disk head, solid state 488 and 561 nm lasers, an iXon Ultra EMCCD (Andor) camera, and a 100 × 1.49 numerical apperture total internal reflection fluorescence objective. Data for patch number/density calculations were collected on a Nikon Ti2-E microscope equipped with a Nikon 60 × 1.49 numerical apperture oil objective and an additional 1.5× magnification, a Yokogawa CSU-W1 spinning disk, and a Photometrics Prime BSI Scientific CMOS camera. For Fim1-mCherry patch density analysis, samples were excited sequentially with 488 nm and 561 nm laser light for 200 ms exposure, and Z-stacks of 7 μm were collected in 0.2 μm steps using NISElements software (Nikon). Three dimensional projections shown in Figures 5*A* and 6*A* were generated with the Fimbrin-mCherry channel data using Imaris software v. 9.5.0 (Bitplane). For mGFP-Wsp1 lifetime analysis, 5 central z-slices were acquired in each cell at 2 s intervals for 150 frames using the 488 nm laser with 8 mW laser power and 350 ms exposure. All images were collected at 25 °C.

### Image analysis

Patch density quantification was performed in Imaris. Typically, more than three clusters of cells were imaged per field of view. Thus, each cluster was cropped in individual images, and a 3D volume reconstruction was rendered using the green channel dataset (mGFP-Wsp1) to obtain the entire volume of the cells. The number of patches was then determined using the spots module utilizing the red channel (Fimbrin-mCherry). To determine the rate of initiation of new endocytic actin patches, all Fim1-mCherry puncta initiated in each cell measured were manually tracked through the first 30 frames of a maximum or sum projection of the video. To determine the mGFP-Wsp1 lifetime, patches were tracked automatically with an Image J actin patch tracking plugin written by Julien Berro (http://campuspress.yale.edu/berrolab/publications/software/) (49). Automatically selected patch trajectories were manually inspected to ensure that the entire lifetime of the event had been captured.

## Data availability

All data are contained within the article.

## Supporting information

This article contains supporting information.

## Conflict of interest

The authors declare that they have no conflicts of interest with the contents of this article.

## Supporting information

supporting_info_figures.Docx

supporting_info_tables
