# Supplementary material for: Analysis of functional surfaces on the actin nucleation promoting factor Dip1 required for Arp2/3 complex activation and endocytic actin network assembly
Source: J Biol Chem. 2022 May 6;298(6):102019. doi: 10.1016/j.jbc.2022.102019 (PMC9168731; doi:10.1016/j.jbc.2022.102019)
Supplement: supporting_info_figures.Docx [file mmc3.docx]

**Supporting Information**

**
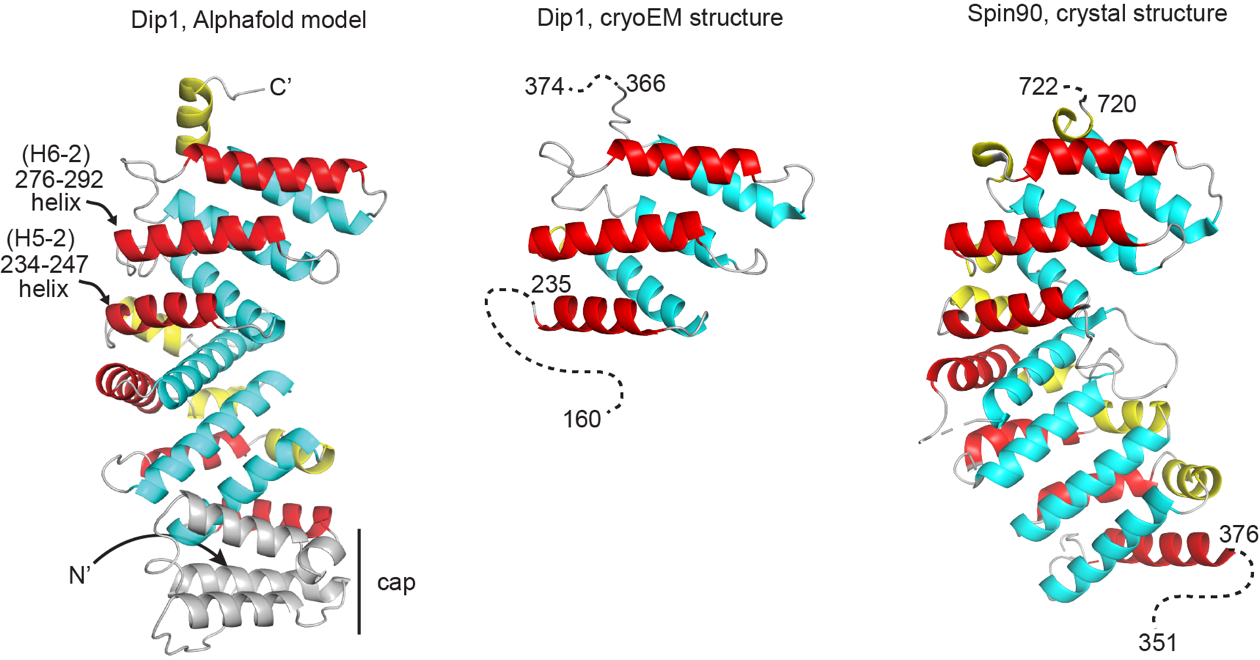
**

**Figure S1**: **Comparison of a model of full length Dip1 generated by Alphafold with other available WDS protein structures.** The left panel shows the Alphafold model of full length Dip1. The middle panel shows the ordered portion of Dip1 from the cryo-EM structure of Dip1 bound to activated Arp2/3 complex (6W17). The right panel shows the structure of a fragment of SPIN90, a human WDS protein (6DED).


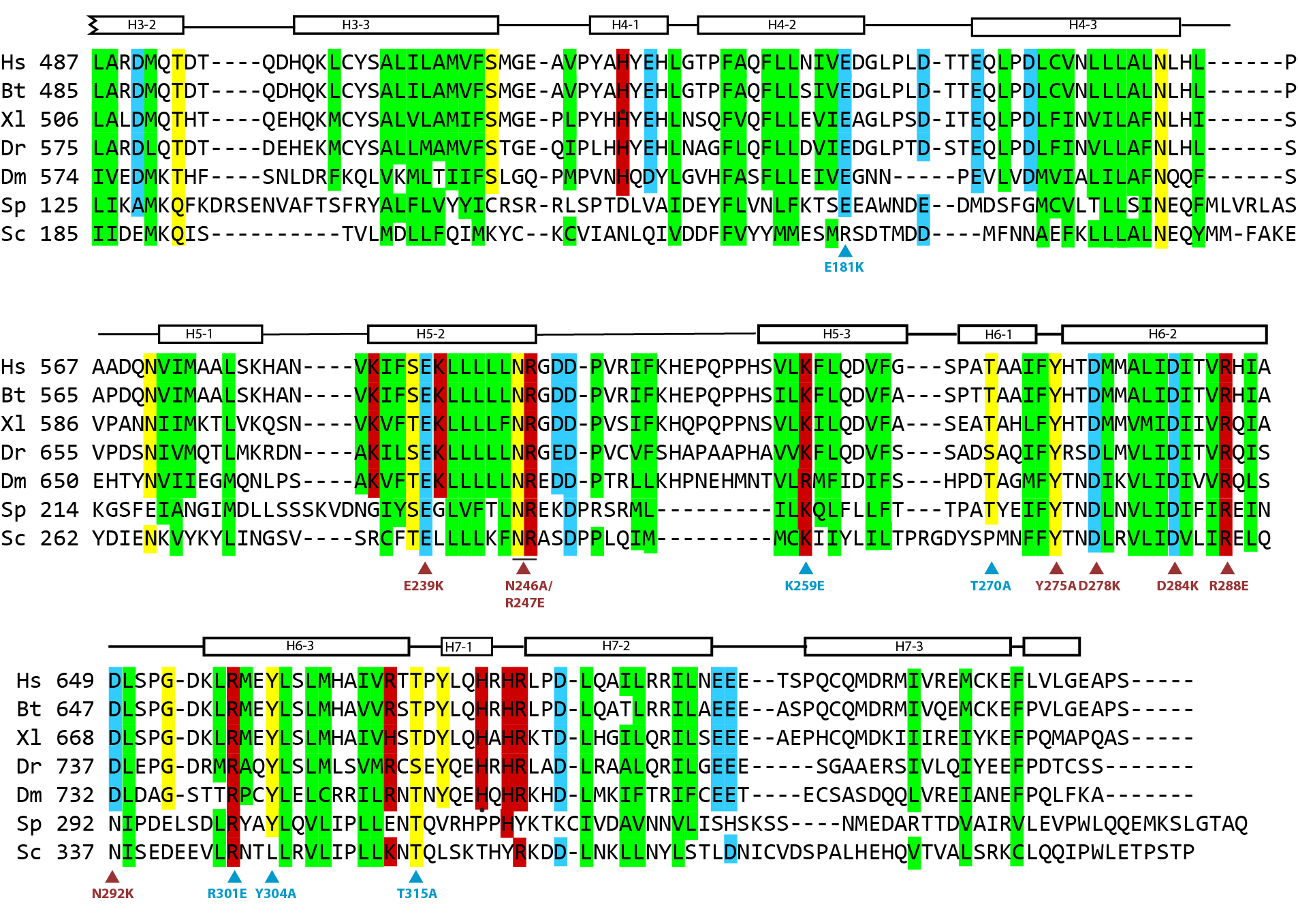


**Figure S2**: **Sequence alignment of the C-terminal portion of WDS proteins from yeasts and metazoans.** Conserved hydrophobic (green), basic (red), acidic (blue), and polar, uncharged (yellow) residues are highlighted. Residues mutated in this study are marked with burgundy (group A) or cyan (group B) arrowheads.


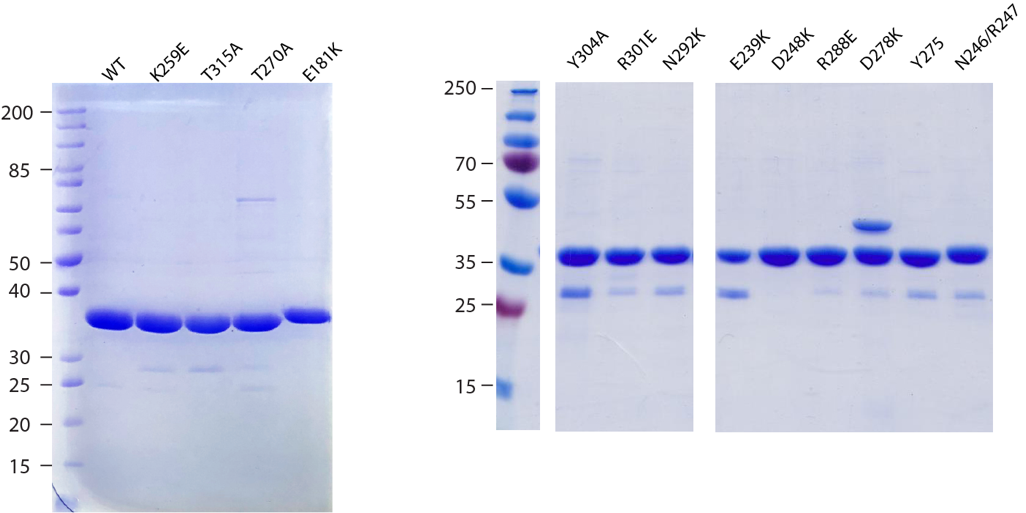


**Figure S3:** **SDS-PAGE of purified wild type and mutant Dip1.** Most preparations included a small amount of GST (~26kD), which was cleaved but not fully separated in some preparations.


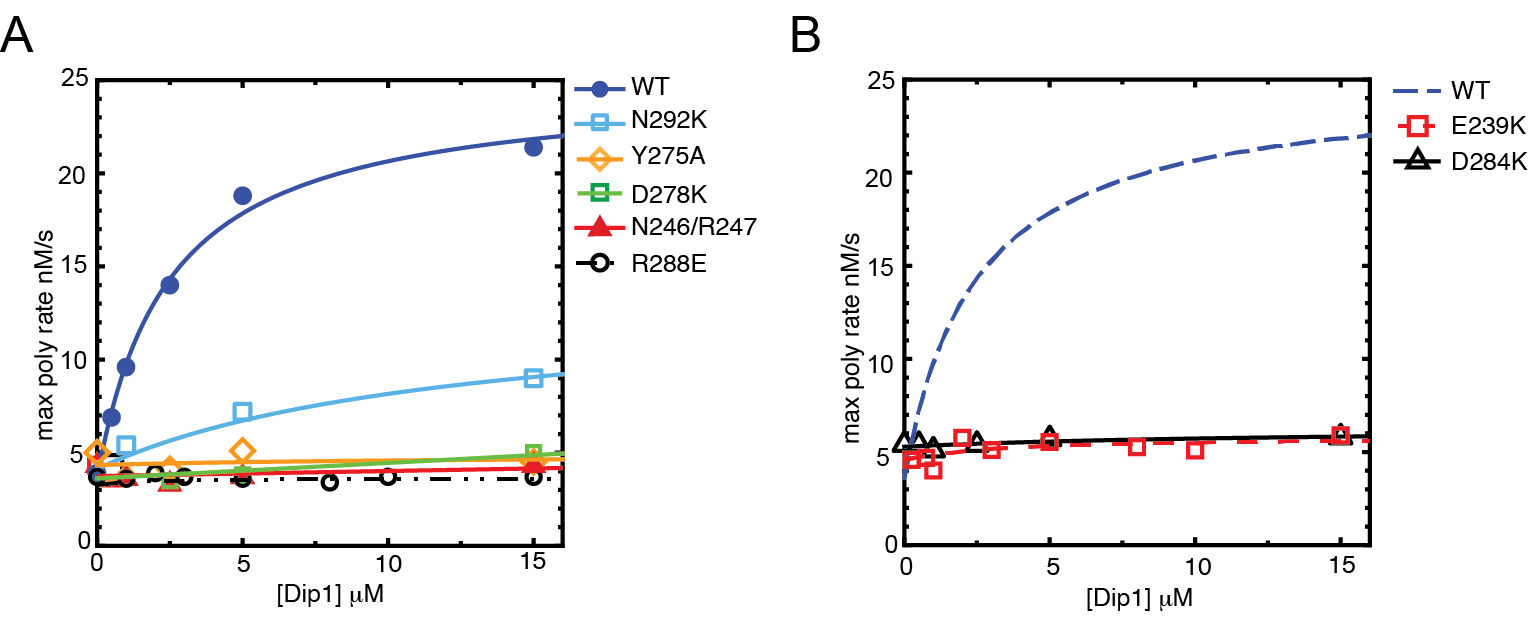


**Figure S4:** **Maximum polymerization rate versus concentration of wild type and group A Dip1 mutants**. *A,* Maximum polymerization rates of a subset of group A mutants calculated from time courses of pyrene actin polymerization in reactions containing 3 μM 15% pyrene labeled actin, 50 nM SpArp2/3 and 0-15 μM Dip1. *B,* Maximum polymerization rates of a subset of group A mutants calculated as described for panel A.


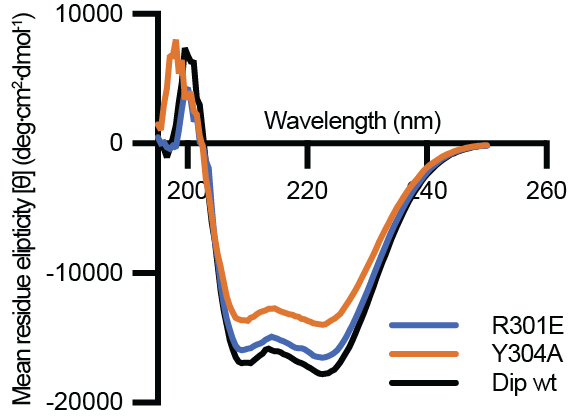


**Figure S5:** **Circular dichroism spectra of wild type, R301E, and Y304E Dip1 mutants.**

**Video S1:** **Hypothetical structural model of full length Dip1 bound to activated Arp2/3 complex.** Full length Dip1 was docked onto the C-terminal fragment of Dip1 in the recently reported structure of Dip1-activated Arp2/3 complex. Note that the N-terminal portion of Dip1 projects away from the clamp subunits (ARPC2 and ARPC4).

**Video S2:** **Structural morph from Dip1 bound to inactive to activated conformations of Arp2/3 complex**. Morph was constructed using ChimeraX. Inactive structure was made by docking Dip1 from Alphafold model onto a homology model of *S. pombe* Arp2/3 complex made with inactive Bos taurus Arp2/3 complex as a template (4JD2). Active structure was taken from cryo-EM structure of Dip1 bound to activated Arp2/3 complex (6W17).
