# Supplementary material for: Analysis of functional surfaces on the actin nucleation promoting factor Dip1 required for Arp2/3 complex activation and endocytic actin network assembly
Source: J Biol Chem. 2022 May 6;298(6):102019. doi: 10.1016/j.jbc.2022.102019 (PMC9168731; doi:10.1016/j.jbc.2022.102019)
Supplement: supporting_info_tables [file mmc4.docx]

**Table S1**: *S. pombe* strains used in this study

| **strain** | **genotype** | **source** |
| --- | --- | --- |
| VS1124A | *h+ ade6-M210 leu1-32 his3-D1 ura4-D18 KanMX6-Pwsp1-mGFP-wsp1 fim1-mCherry-natMX6* | V. Sirotkin |
| QL002 | *h+ ade6-M210 leu1-32 his3-D1 ura4-D18 KanMX6-Pwsp1-mGFP-wsp1 fim1-mCherry-natMX6 dip1∆::ura4* | This study |
| QL004 | *h+ ade6-M210 leu1-32 his3-D1 ura4-D18 KanMX6-Pwsp1-mGFP-wsp1 fim1-mCherry-natMX6 dip1∆::****dip1R288E*** | This study |
| QL005 | *h+ ade6-M210 leu1-32 his3-D1 ura4-D18 KanMX6-Pwsp1-mGFP-wsp1 fim1-mCherry-natMX6 dip1∆::dip1****R301E*** | This study |
| QL006 | *h+ ade6-M210 leu1-32 his3-D1 ura4-D18 KanMX6-Pwsp1-mGFP-wsp1 fim1-mCherry-natMX6 dip1∆::dip1****E239K*** | This study |
| QL007 | *h+ ade6-M210 leu1-32 his3-D1 ura4-D18 KanMX6-Pwsp1-mGFP-wsp1 fim1-mCherry-natMX6 dip1∆::dip1****K259E*** | This study |
| SpBN169 | *h+ ade6-M210 leu1-32 his3-D1 ura4-D18 KanMX6-Pwsp1-mGFP-wsp1 fim1-mCherry-natMX6 dip1∆::dip1****N292K*** | This study |
| SpBN173 | *h+ ade6-M210 leu1-32 his3-D1 ura4-D18 KanMX6-Pwsp1-mGFP-wsp1 fim1-mCherry-natMX6 dip1∆::dip1****E181K*** | This study |
| SpBN174 | *h+ ade6-M210 leu1-32 his3-D1 ura4-D18 KanMX6-Pwsp1-mGFP-wsp1 fim1-mCherry-cloNAT dip1∆::dip1****D278K*** | This study |
| SpBN175 | *h+ ade6-M210 leu1-32 his3-D1 ura4-D18 KanMX6-Pwsp1-mGFP-wsp1 fim1-mCherry-cloNAT dip1∆::dip1****Y275A*** | This study |
